# On drug treatment and social control: Russian narcology's great leap backwards

**DOI:** 10.1186/1477-7517-5-23

**Published:** 2008-06-24

**Authors:** Richard Elovich, Ernest Drucker

**Affiliations:** 1 Columbia University, Mailman School of Public Health, NYC, USA; 2 Montefiore Medical Center, Albert Einstein College of Medicine, NYC, USA

## Abstract

The medical discipline of narcology in Russia is a subspecialty of psychiatry from the Soviet era and it is given warrant to define the scope of health activities with regard to alcohol and other drug use, drug users, and related problems. Narcological practice is in turn constrained by the State. The emergence of widespread injection opiate use and associated HIV morbidities and mortalities during the first decade following the collapse of the Soviet Union has brought the contradictions in Russian narcological discourse into high relief. Narcology officials in the Russian Federation have consistently opposed substitution treatment for opiate dependence – the replacement of a short-acting illegal substance with a longer acting prescribed drug with similar pharmacological action but lower degree of risk. Thus, despite the addition of methadone and buprenorphine to WHO's list of essential medicines in 2005 and multiple position papers by international experts calling for substitution treatment as a critical element in the response to HIV (IOM, 2006; UNODC, UNAIDS, and WHO, 2005), methadone or buprenorphine remain prohibited by law in Russia.

The authors detail Russian opposition to the prescription of methadone and buprenorphine, describing four phenomena: (1) the dominance of law enforcement and drug control policy over public health and medical ethics ; (2) the conflation of Soviet era alcoholism treatment with treatment for opiate dependence; (3) the near universal representation of detoxification from drugs as treatment for dependence; and (4) a framework for judging treatment efficacy that is restricted to "cure" versus "failure to cure," and does not admit its poor outcomes or recognize alternative frameworks for gauging treatment of opiate dependence. In keeping with this position, Russian narcology officials have taken an implacable ideological stance toward illicit drug use, the people who use drugs, and their treatment. By adopting policies and practices totally unsupported by scientific evidence and inquiry, officials in Russia have rendered narcology ( and medical practice) insensitive to the alarming rates and continued spread of HIV, with its dire morbidity and mortality rates in the Russian Federation, turning their backs on all the other health problems posed by opiate use and dependence itself.

## Editorial

The spring of 2008 marks the opening of a year of reflection meant to culminate in a high-level United Nations meeting assessing progress since the 1998 UN General Assembly Special Session on Drugs. Whatever member states declare about the international drug control regime, it is clear that in Russia we are bearing witness to one of the catastrophes in the history of HIV – the lack of response to the epidemic in Russia. In particular, we must point to the special responsibility that Russian medical and public health officials bear for creating and sustaining this deadly situation. The roots of the problem lie in their basic position on the problem of drug use and addiction – a rejection both of the core principles of harm reduction and of the usual obligations of medicine (as expressed in the Hippocratic oath) to "first do no harm". Indeed, the leaders of the sub-discipline of Russian psychiatry known as "narcology" seem to assume no special obligation to save lives, offering substandard treatments and insisting on their value. As a consequence, treatment for opiate dependence serves the end of social control and enforcement, but they do little to treat addiction.

An arm of the state, narcological dispensaries are scattered throughout Russia. These offices are structured primarily to provide detoxification for opiate users and alcoholics, and most provide few or no harm reduction interventions to reduce HIV and hepatitis among users. The academic leaders of narcology officially determine the scope of public health and risk reduction interventions and decide which are to be regarded as effective. Despite the addition of methadone and buprenorphine to WHO's essential medicines list, Russia outlaws any form of substitution treatment. These failures of narcology can be seen as an engine driving the HIV epidemic in Russia and, through its omissions, commissions, gaps, and blockages – making it worse each day.

With nearly one million HIV infections, some 80 percent of which are related to the sharing of drug injection equipment, Russia has the fastest growing HIV epidemic in the world.

Despite their proven efficacy, syringe availability, ready access to methadone and buprenorphine for maintenance, or effective social support programs for drug users all remains very limited in Russia. In contrast to the United States, it is methadone that remains the most contested of harm reduction interventions in Russia. *"The misguided practice of issuing addicts a "narcotic ration" was long ago prohibited*. [1:253]"declared Edward Babayan, a pioneer of narcology in the former Soviet Union, in a text book for psychiatrists regarded as primary in the field. This statement is the only reference in the entire textbook to drug substitution treatment, and no history is provided of the approach before it was prohibited, nor any explanation or evidence for terming it a "misguided practice" nor. As with narcological pronouncements more generally in Russia, the hierarchical status of the author substitutes for science.

Consistent evidence from around the world shows that opiate dependence treatments work most effectively when they widen from an exclusive goal of abstinence and seek to foster multiple outcomes – including reduction in use of illicit opiates, reduction in injections and exposures to blood-borne infections such as HIV and hepatitis, reduction in drug overdoses, better management of existing health problems, and improvement of normative social functioning. While some 800,000 patients now have access to methadone and buprenorphine, the position of many narcologists in Russia is that the world is turning away from the medications, indicating Russian reluctance to implement them.

Vladimir Mendelevich, a Russian psychiatrist who has actively critiqued the dominant narcological model, reported at a 2006 satellite meeting of the UN Commission on Narcotic Drugs (UN CND) findings from his recent research [2] that: (1) the majority of narcologists in Russia offer nothing but heavily medicalized detoxification; (2) the majority of patients relapse within six months, and (3) the majority of narcologists are satisfied with the field and do not think major changes are required. Echoing the declarations of Edward Babayan (2001) and Nikolai Ivanets (1998), now head of a leading Russian narcological institution, the discipline in Russia has consistently aligned itself with the restrictive and punitive. Babayan was the author of the infamous "drug table" that subjected those in possession of the residue in a used syringe to years of imprisonment, and those possessing as little as a single dose of heroin liable to still longer incarceration. In this context, resistance of Russian addiction professionals to substitution treatment can be seen as expressing the underlying conviction that illicit drug users are a criminal class that needs to be put under control, and if necessary, isolation.

The approach that regards addicts as criminals is based on a number of categorical assumptions, unsupported by empirical data: (1) the patient does not realize his social and health danger; (2) the patient does not completely understand the character of his own activity; (3) the patient cannot control it; (4) the patient brings harm to himself and his surroundings [3]. In the circular thinking that confounds clinical practice with law enforcement, any user of a narcotic that he or she was not prescribed by a physician is an abuser likely to cause harm to self or others [1,4].

These attitudes and policies have much in common with Federal policies in the US. Like Russia, the US emphasizes criminalization as the default response to the problem of addiction and the vast majority of public resources are directed at arrest, prosecution, and incarceration of drug users – not treatment. And, as in the US, mass incarceration of drug users in Russia, under brutal conditions, produces a set of predictable adverse results for the individuals affected, and enables the continued spread of HIV throughout the general population.

An example of the dedication of Russian narcology to reinforcing its positions can be found in an official memorandum attacking methadone treatment – written and widely distributed by Ivanets and other prominent Russian narcologists. The memorandum urges that Russians say "NO TO METHADONE PROGRAMS IN THE RUSSIAN FEDERATION". For the authors of the memorandum, who include V. N. Krasnov Professor, Chair, Russian Society of Psychiatrists; N. N. Ivanets, Professor, Director, National Center on Addictions, Member-correspondent of the Russian Academy of Medical Sciences; and T. B. Dmitrieva, Professor, Deputy Chair of the Russian Society of Psychiatrists, "The effective way to solve the problem of drug addiction treatment is an intensive search for and introduction of new methods and means that focus on complete cessation of drugs use by patients with addiction, their socialization into a new life style free from drugs, but not on exchanging from one drug to another." While Russian drug users and their families wait for the coming of this millennium, HIV continues its march across Russia unabated.

The authors construct a xenophobic edifice that makes methadone appear as a plot against Russia. "Foreign emissaries have increasingly raised the issue of introducing substitute therapy in the form of methadone programs for treatment of patients with heroin drug addiction". By presenting themselves as having the moral authority to know what is best for drug addicts, and by representing their assertions as scientific facts, these experts regard substitution treatment as if it were an "foreign enemy at the gate". Particularly alarming is the fact the Dr. Dimitrieva, the memo's last author, is also a Member of the International Narcotics Control Board – the independent, "quasi-judiciary" body responsible for setting standards and policing compliance with international drug treaties at the United Nations. In this instance, judgment appears to have been rendered without evidence.

There have been many letters sent to the Russians from outside public health and medical officials and other experts including a report on the raft of evidence about methadone substitution, "Say Yes to Methadone and Buprenorphine in the Russian Federation" by Icro Maremmani and colleagues from the European Opiate Addiction treatment Association and its US counterpart (AATOD). In addition, international experts have prepared a report providing a point-by-point refutation of the memos' many errors of commission and omission. (attached in English and Russian). But these seem to fall on deaf ears in Russia.

If Russia is nearly alone in opposition to such therapies, this is seen as a point of nationalist pride. In stark contrast with other medical disciplines, narcology has not only stopped its progress, but has regressed towards what Mendelevich argues is a pseudo-science *vis a vis* assumptions about the nature of opioid dependence, characteristics of drug users, and the character of dependence and acceptable treatment. Mendelevich cites a 2003 appeal by "Orthodox Doctors of Moscow" that was entitled "Stop Depravity," in which the authors representing the "medical view" of the Orthodox Russian Church demanded:

Methadone being a toxic drug with a significant euphoric effect can quickly cause severe addiction. Thus, not solving the problem of heroin addiction, it can become widely spread in the case that it is recommended as treatment. The program of methadone substitution therapy will generally become the first step to legalization of drugs in Russia, and we demand on not admitting its ratification by the Lower House of Russian Parliament.

[5,6]

As with the U.S., Russia's influence extends beyond its borders. Other republics of the former Soviet Union (FSU) adopt Russian narcology as their own model, or exist in uneasy tension with efforts to depart from the Russian model.

What is offered as drug addiction instead? Despite widespread opiate use and its direct association with increases in HIV incidence in Russia, detoxification or 'blood purification,' and psychotherapies geared to late stages of alcoholism are largely the only available narcological treatment option for opiate dependence.

This may be best illustrated with the example of *codirrovanir* or encoding, where patients undergo a hypnoid based therapy worthy of Rasputin. A series of misrepresentations, including a signed consent letter is witnessed by family members. Patients are manipulated into believing that if they drink during a prescribed period of time, they will die [7]. This approach is among the most prevalent form of psychotherapy practiced by Russian narcologists: one survey conducted among patients and their relatives indicates that coding accounted for up to 80% of methods offered as psychotherapy. There is a financial incentive to this approach: as Mendelevich notes, encoding is not included in Russian narcology's list of free medico-psychological curative-rehabilitative services, so patients must pay[5]

Limited access to current international research and travel opportunities for narcologists is a key element of the problem. In much of the former Soviet Union, narcologists who have come into the field since the emergence of injection drug use and HIV consistently report a lack of advanced educational and clinical training to meet the challenges they face. Some motivated narcologists have educated themselves beyond the 'received knowledge' available through night shift internships in a dispensary, and describe a growing dissatisfaction with the vertical relationship in which patients are regarded as bit players in their own recovery. A few narcologists might be characterized as early adopters – they use the internet to break their isolation, attend international meetings, and are initiating reforms within a narcological dispensary or outside in an NGO [8].

For the majority, however, emphasis on administrative duties and low salaries reduces incentives for narcologists to change their approach to that of a care giver. In the words of a Ukrainian physician who also works in Russia [8]:

The narcologist is not able or allowed to understand him or herself primarily as a caregiver or a caregiver at all. You get the position, two years after medical school, and then you withdraw from care; you understand that your way is to be in business for yourself where you get money for certifying people [as being free from drug use] or for detox medications. This is the main issue of the degradation of the doctor: when he is deprived of up-to-date education, when he is relegated to the status of bureaucrat policeman, and when his reaction to new ideas about treatments, rather than based on science, are based on rumor, like he is just a guy on the street. I feel myself standing not in a hospital or professional setting, even with the white coats, but in a bazaar.

Those narcologists who push back or are innovative are often seen as trouble makers and are vulnerable to rumors and scandal to invalidate their work. A recent essay in Lancet describes how a website Mendelevich set up to open up a "scholarly debate" on the use of methadone resulted in visits from prosecutors and state drug control for "propagandizing of narcotics," and was requested to visit the Prosecutor's office. [9].

As a step toward critical examination of where the criminal framework obscures that of public health, HRJ is pleased to now publish Lev Levinson's report " Half a Gram – A Thousand Lives". Focusing on the weight of drugs confiscated from users and how these are mechanistically linked to criminal penalties, in a pattern similar to those employed in the US beginning with New York's Rockefeller Drug Laws in 1973, Levinson illuminates the larger question of why criminal penalties and social control have so overshadowed other approaches. Despite legal reforms hailed as steps forward because they reduced criminal penalties, Levinson

Notes that fundamental contradictions in what is considered evidence remain: mandatory sentences are determined by the weight of drugs seized from individuals – REGARDLESS OF PURITY. Thus a dealer with 2 grams of 80% pure heroin faces the same penalties as the user who has 2 grams of cut drug that's only 20% pure. As Levinson notes, "*...For acts not involving sale (acquisition, possession, transportation, production, or processing), the amount of the substance involved in the act is the sole determinant of whether the perpetrator is criminally prosecuted or is subject only to administrative punishment in the form of a fine of up to 1000 rubles or 15 days of detention."*

As is the case everywhere, most heroin addicts' lives in Russia are bound up with buying and selling illicit drugs, making them easy prey for arrests. They are then characterized as "drug dealers", and prosecuted accordingly. Levinson notes that "*According to the revision of article 228*^1 ^*of the Criminal Code* ... the *sale of narcotics, like their production for purposes of distribution, is a criminal offense regardless of amount involved ... considered a felony and is punishable by incarceration for four to eight years"*.

The new revisions to Russian law, Levinson notes, have *"become a survival issue for the Federal Service to Control Narcotics trafficking"*, whose careers and personal fortunes are closely linked to keeping criminal pressure on addicts. Indeed, the federal drug control service issues glowing reports of its interdiction efforts each year to the international community, and no doubt will seek to have their successes reckoned as the world reflects on progress in the war on drugs. If past is prologue, unmentioned will be the ways in which these policies spell more infection and early death for drug users who remain at great risk not only for police abuses, but for blood-borne infections that have already claimed too many Russian lives.
